# Quantifying Exocytosis by Combination of Membrane Capacitance Measurements and Total Internal Reflection Fluorescence Microscopy in Chromaffin Cells

**DOI:** 10.1371/journal.pone.0000505

**Published:** 2007-06-06

**Authors:** Ute Becherer, Mathias Pasche, Shahira Nofal, Detlef Hof, Ulf Matti, Jens Rettig

**Affiliations:** Universität des Saarlandes, Physiologisches Institut, Homburg, Saar, Germany; Medical College of Georgia, United States of America

## Abstract

Total internal reflection fluorescence microscopy (TIRF-Microscopy) allows the observation of individual secretory vesicles in real-time during exocytosis. In contrast to electrophysiological methods, such as membrane capacitance recording or carbon fiber amperometry, TIRF-Microscopy also enables the observation of vesicles as they reside close to the plasma membrane prior to fusion. However, TIRF-Microscopy is limited to the visualization of vesicles that are located near the membrane attached to the glass coverslip on which the cell grows. This has raised concerns as to whether exocytosis measured with TIRF-Microscopy is comparable to global secretion of the cell measured with membrane capacitance recording. Here we address this concern by combining TIRF-Microscopy and membrane capacitance recording to quantify exocytosis from adrenal chromaffin cells. We found that secretion measured with TIRF-Microscopy is representative of the overall secretion of the cells, thereby validating for the first time the TIRF method as a measure of secretion. Furthermore, the combination of these two techniques provides a new tool for investigating the molecular mechanism of synaptic transmission with combined electrophysiological and imaging techniques.

## Introduction

Ca^2+^-dependent exocytosis of synaptic vesicles is preceded by the anchoring of the vesicles to the plasma membrane in a docking step and by a membrane-delimited maturation step called priming. These different molecular states of vesicles might be identified electrophysiologically by the rate at which they fuse with the plasma membrane. In chromaffin cells a stepwise increase of [Ca^2+^]_i_ leads to two kinetic components of secretion, a burst phase and a sustained component as measured by membrane capacitance increase. Vesicles fusing during the burst phase are presumably primed vesicles. Docked vesicles which are primed while [Ca^2+^]_i_ remains high are released during the sustained component [1], [2]. As membrane capacitance recording measures only the increase in surface area upon fusion of vesicles with the plasma membrane [3], estimates of priming and docking with this method are indirect. In contrast, total internal reflection fluorescent microscopy (TIRF-Microscopy) enables selective visualization of synaptic vesicles or large dense core vesicles (LDCVs) at the membrane close to the glass/cell interface, i.e. at the footprint of the cell [4]–[12]. Therefore, TIRF-Microscopy provides an ideal tool to observe docking and priming directly [13], [14]. However, whether secretion at the footprint of the cell is representative of secretion over the entire cell surface is a matter of debate.

In this study, we performed for the first time simultaneous capacitance and TIRF-Microscopy measurements on bovine adrenal chromaffin cells and investigated vesicle secretion under different physiological conditions. We find that secretion at the footprint of the cells is qualitatively and quantitatively correlated with the overall secretion of the cell demonstrating that TIRF-Microscopy is well suited to study the docking and priming reactions of LDCVs.

## Results

### Exocytosis of the entire cell correlates with the secretion at the footprint

**Figure 1:**
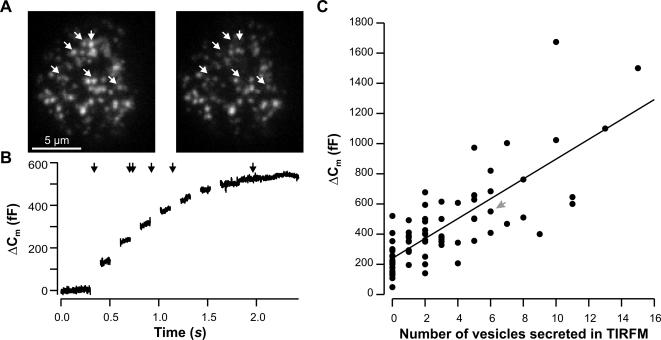
TIRF-Microscopy measurements of exocytosis correlate with simultaneous cell capacitance measurements. A. TIRF-Microscopy image of the footprint of a representative chromaffin cell transfected with NPY-mRFP taken prior to (left) and immediately after (right) depolarization (seven 100 ms depolarizations to −5 mV at 5 Hz). White arrows indicate vesicles that were secreted during stimulation. B. Corresponding membrane capacitance recording of the cell shown in (A). Black arrows indicate the approximate time of secretion in TIRF. C. A high correlation (p<0.001, correlation coefficient 0.76, n = 77) was observed between the number of secreted vesicles measured by TIRF-Microscopy and the corresponding capacitance increase. Gray arrow points to the exemplary cell shown in A and B.

In order to determine the relationship between vesicle fusion assessed by membrane capacitance increases and that visualized by TIRF-Microscopy, we patched single bovine chromaffin cells in which LDCVs had been labeled with Neuropeptide Y (NPY) coupled to the monomeric red fluorescent protein (mRFP) [15]. During whole-cell recording cells were imaged at 5 Hz and exocytosis was triggered by depolarizing pulses (see Methods). Figure 1A shows the footprint of a representative cell immediately before and after secretion was induced by depolarization (see Supplementary Movie S1). While secretion of six vesicles could be observed at the footprint (Figure 1A, arrows), the simultaneously recorded capacitance increase was 527 fF (Figure 1B). The time course of secretion was similar in both measurements as can be seen by the arrows in figure 1B marking the approximate time of secretion detected in TIRF-Microscopy. The results from 19 cells stimulated 1 to 4 times showed that exocytosis as indicated by membrane capacitance increase is linearly correlated to exocytosis observed using TIRF-Microscopy (Figure 1C; correlation coefficient 0.76, p<0.001, n = 77.

### Enhancing the detection of secretory events in TIRF-Microscopy improved the correlation between exocytosis measured by TIRF-Microscopy and by membrane capacitance recording

To determine whether the number of vesicles secreted at the footprint is proportional to the number of LDCVs secreted over the entire cell membrane, we compared the ratio of the surface area of the footprint to the entire cell surface to the ratio of the number of vesicles secreted at the footprint to the global increase of membrane capacitance. The mean surface area of the footprint of the cells was 98.94±4.42 µm^2^ and their average membrane capacitance was 8.19±0.21 pF, which corresponds to an average total surface area of 819±21 µm^2^ (assuming 1 µF·cm^−2^, Table 1, [16]). Hence, the footprint area represented 12±1% of the entire cell surface. Further, mean secretion detected by TIRF-Microscopy was 3.2±0.4 vesicles, corresponding to a capacitance increase of 6.0±0.8 fF if the membrane capacitance of one vesicle is 1.9 fF [17]. The average, global capacitance increase was 447±33 fF. Thus, secretion at the footprint represented only 1.1±0.1% of global secretion of the cells. From these numbers we can conclude that secretion visualized at the footprint was about 10 times lower than secretion measured over the entire cell surface by capacitance.

**Table 1:** Comparison of exocytosis measured with TIRF-Microscopy using various methods to label the LDCVs or to elicit secretion.

Label Stimulation	mRFP Depolarization	Venus Depolarization	mRFP Flash photolysis
**Footprint area**	98.94±4.42 µm^2^	70.14±3.00 µm^2^	70.89±4.53 µm^2^
**Total plasma membrane surface area (1)**	8.19±0.21 pF	6.56±0.25 pF	6.75±0.19 pF
	819±21 µm^2^	856±25 µm^2^	675±19 µm^2^
**Footprint/Total area**	**12.4±0.5%**	**10.8±0.4%**	**10.5±0.5%**
**Secretion at footprint (2)**	3.2±0.4 vesicles	2.5±0.3 vesicles	1.8±0.2 vesicles
	6.0±0.7 fF	4.7±0.6 fF	3.4±0.4 fF
**Global secretion**	446±33 fF	282±26 fF	242±22 fF
**Footprint/global secretion**	**1.1±0.1%**	**1.7±0.2%**	**1.8±0.2%**
**n**	77	54	65

**Conversion factors :** (1) 1 µF·cm^−2^ [16], (2) 1.9 fF ·vesicle^−1^ [17]

**Figure 2:**
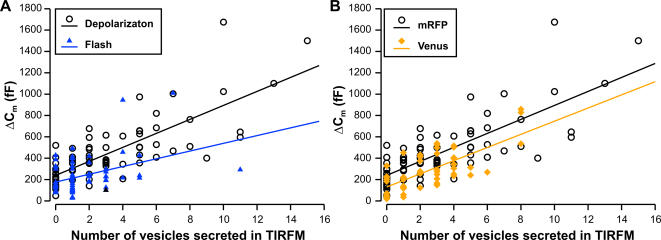
Different triggers and different vesicle labels lead to better correlates between exocytosis measured by TIRF-Microscopy and by membrane capacitance recording. A. Correlation plot of exocytosis measured by TIRF-Microscopy and by membrane capacitance recording when secretion was either induced by a depolarization train (open circles) or by flash photolysis of caged Ca^2+^ (blue triangles). B. Correlation plot of exocytosis measured by TIRF-Microscopy and by membrane capacitance recording. LDCVs were either labeled by overexpretion of NPY-mRFP (open circles) or of NPY-Venus (yellow diamonds).

The discrepancy between the overall secretion measured by membrane capacitance and secretion measured at the footprint of the cell measured by TIRF-Microscopy can be readily explained. First, it is likely that not all LDCVs are loaded with NPY-mRFP. Steyer and Almers [9] showed that the density of docked vesicles is about 1.7 µm^−2^ using electron microscopy and that about 1.3 vesicles·µm^−2^ were observed with TIRF-Microscopy using acridine orange labeling of LDCVs. This density is about twice that of NPY-mRFP labeled vesicles that we observed (0.61±0.02 vesicles·µm^−2^). Second, it is possible that diffusion of Ca^2+^ between membrane and glass coverslip is hindered hence, that the Ca^2+^ concentration in microdomains at the footprint of the cell is reduced as compared to the concentration reached in microdomains next to the membrane of the rest of the cell. We tested this hypothesis by inducing secretion using flash photolysis of caged Ca^2+^. This method allowed us to raise [Ca^2+^]_i_ homogenously in the cell in a stepwise manner. We found that the number of vesicles secreted at the footprint of the cell varied strongly from one cell to another (Figure 2A). If we compare the ratio between secretion at the footprint of the cell and the overall secretion of the cell we find that in average we measure 1.8±0.2% (n = 65) of the global secretion with TIRF-Microscopy when secretion was induced by flash photolysis of caged Ca^2+^. This represents 30% more exocytotic events than were observed when secretion was induced by depolarization (Table 1). Due to high cell to cell variability this increase of secretion measured by TIRF-Microscopy was not significant. Finally it is likely that we do not identify all secretory events that occur at the footprint of the cell. Exocytosis of a vesicle was visualized by a rapid loss of its fluorescence (in less than 200 ms). Many vesicles disappear more slowly and our interpretation of a slow loss of fluorescence was that a vesicle detaches from the membrane and moves back to the cytoplasm. However, it has been shown using carbon fiber amperometry that the opening of the fusion pore can be slow, lasting several hundreds of milliseconds [18]. Such a fusion event would probably result in a slow loss of fluorescence [19] and we would not identify it as exocytosis. We tested this hypothesis using another fluorescent tag of NPY. Instead of mRFP we fused NPY to Venus. This yellow variant of eGFP is pH sensitive and signals the fusion pore opening by a bright fluorescent flash in response to the neutralization of the lumen of the vesicle [20], [21]. With this fluorescent marker we found that the correlation coefficient (0.65, p<0.001, n = 54, Figure 2B) between exocytosis measured with membrane capacitance and TIRF-Microscopy was similar to that when the vesicles were marked with NPY-mRFP. However, about one third of the fusion events visualized by TIRF-Microscopy were missed using mRFP in comparison to Venus to label the LDCVs (p = 0.039, n = 54, Table 1).

### Cells secrete with identical time course at the footprint and over their entire surface

**Figure 3:**
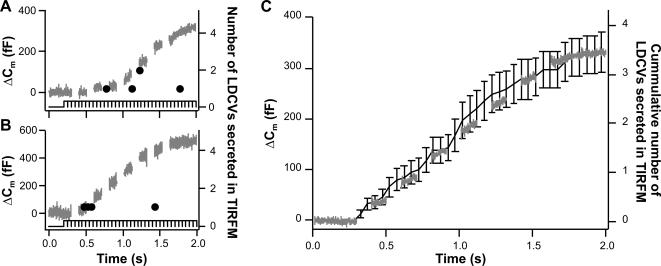
The time course of secretion at the footprint of the cell correlates with the time course of secretion over the entire cell surface. A. and B. Secretion from two exemplary cells using membrane capacitance recording (gray line, left axis) and TIRF-Microscopy (back dot, right axis). The time of secretion is given by the time at which an image was acquired (black line, bottom of the graphs). C. Average secretion of 6 cells stimulated 1 to 3 times (n = 13). The gray line represents the average membrane capacitance recording and the black line represents the average cumulative secretion visualized by TIRF-Microscopy. Error bars represent S.E.M. Both curves have been scaled so that the starting value and the end value coincide. Note that both curves overlap over the entire time course of recording.

As evident from figure 1B, the time course of secretion detected as membrane capacitance increase and that detected using TIRF-Microscopy appeared similar. However, due to low sampling rate and lack of synchronization of electrophysiological recording and imaging, the measurements were not accurate enough to determine a correlation of the time courses of secretion in both methods. We repeated the experiment, increasing the sampling rate to 20 Hz and synchronizing the electrophysiological measurements with the imaging by recording the ON/OFF signal of the camera with the electrophysiological measurements. Figure 3 (A and B) shows the recording of two exemplary cells whereby the black line represents the acquisition state of the camera and the black dots the secretion of vesicles detected in TIRF-Microscopy. It is clearly visible that secretion at the footprint occurred only when the membrane capacitance was increasing. We averaged the cumulative secretion measured in TIRF-Microscopy of 6 cells that were stimulated one to four times (n = 13) and compared it to the average membrane capacitance recordings (Figure 3C). Since both curves perfectly overlap, we can conclude that the time course of secretion at the footprint correlates with secretion over the entire cell surface.

### Enhancing priming results in increased secretion over the entire cell surface and at the footprint of the cell

**Figure 4:**
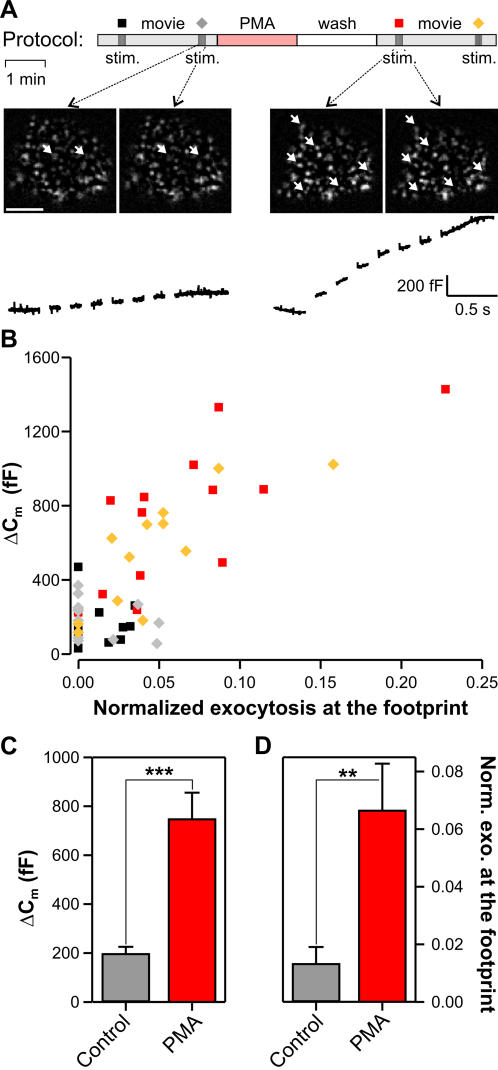
Exocytosis at the cell footprint and on the entire cell surface was increased by a similar factor after PMA treatment. A. The cells were imaged with TIRF-Microscopy and their membrane capacitance was recorded simultaneously for 3 min. During this period secretion was stimulated twice with an interval of 2 min (top). The cells were then treated with 250 nM PMA for 2 min, washed for 2 min and were then stimulated twice. There was a strong increase in secretion as measured by both TIRF-Microscopy (middle) and by membrane capacitance change (bottom) after PMA application (right). Scale bar represents 5 µm. B. The correlation plot shows that exocytosis in response to both stimuli of the same recording period was similar when measured by either TIRF-Microscopy or membrane capacitance recording. A strong increase of secretion was observed upon PMA application using both measurement methods. The number of vesicles secreted at the footprint was normalized to the number of vesicles prior to fusion. Symbols are derived from the protocol above (A, top). C and D. Average membrane capacitance increase (C) and secreted vesicles measured using TIRF-Microscopy (D) upon stimulation immediately prior to and after PMA treatment. Note that exocytosis is increased (p<0.005) by a similar factor at the cell footprint and in the entire cell.

We next examined if modulating global secretion by enhancing priming is reflected by a change of secretion at the footprint of the cell. We applied phorbol myristate acetate (PMA), a phorbol ester that activates both protein kinase C and the priming factor Munc13 [22]–[24]. In chromaffin cells, it was shown previously that PMA leads to a 2–3 fold increase in secretion due to an increase in the releasable pools of vesicles [22]. Secretion was measured in two recording periods of 3 min during which the chromaffin cells were stimulated twice with an interval of 2 min (Figure 4A). Before the second recording period 250 nM PMA was applied to the bath for 2 min, followed by a 2 min wash. Figure 4A depicts a cell secreting 2 vesicles at the footprint prior to PMA application and 6 vesicles after treatment. This increase of secretion at the footprint was associated with an increase of the membrane capacitance from 167 fF to 887 fF. In 12 out of 13 cells, an increase in membrane capacitance was accompanied by an increase of vesicle fusion observed in TIRF-Microscopy (Figure 4B). The 5.0±2.7 fold enhancement of secretion at the footprint (TIRF-Microscopy, Figure 4D) was similar to the 3.7±0.8 times increase of secretion observed for the entire cell using membrane capacitance recording (Figure 4C). In addition, exocytosis over the entire cell surface and exocytosis at the footprint were similar in responses to both stimuli within a same recording period (Figure 4B). We conclude that secretion of LDCVs observed at the footprint of the cell is representative of secretion of the entire cell.

## Discussion

We investigated the relationship between secretion at the footprint of the cell visualized by TIRF-Microscopy and secretion over the entire cell surface measured by membrane capacitance recording. We found that the number of vesicles secreted at the footprint of the cell correlated well with the membrane capacitance increase upon depolarization of the cell. However, we observed that, when corrected for observed surface area, secretion visualized by TIRF-Microscopy is 10 times lower than secretion measured by membrane capacitance recording. This apparent discrepancy can be explained by several factors: 1. Only about half of the LDCVs were loaded with NPY-mRFP; 2. Secretion at the footprint of the cell was somewhat reduced in comparison to the rest of the cell when exocytosis was induced by depolarization as diffusion of Ca^2+^ between membrane and glass coverslip is hindered; 3. A large proportion of exocytotic events occurs with a slow decrease in fluorescent signal which renders their detection more difficult. Note that, due to the small number of secretory events observed at the footprint, exocytosis observed with TIRF-Microcopy will not necessarily be linearly correlated with the exocytosis measured by membrane capacitance recording under conditions in which overall secretion of the cell is low (<200 fF). Instead it becomes a stochastic signal that may be quite different from the mean number of exocytotic events.

We showed that the time course of secretion was identical in both measurement methods and that increasing secretion by PMA treatment resulted in a similar increase of global secretion and of secretion at the footprint of the cell. Taken together our results demonstrate that secretion at the footprint of the cell is a scaled index of the secretion over the entire cell surface. Furthermore, as it has been shown that PMA increases secretion in chromaffin cells primarily by enhancing priming, we can conclude that priming observed at the footprint of the cell is representative for the priming of all LDCVs of the cell.

These data are inconsistent with the hypothesis that modification of the cytoskeleton (i.e. stress fibers) occurring at the adhesion site of the cell to its substrate modifies the behavior of LDCVs [25], [26]. Therefore, we conclude that the mobility of the vesicles observed with TIRF-Microscopy is similar to the mobility of the vesicles elsewhere in the cell and that TIRF-Microscopy is an appropriate tool to study the docking and priming reaction of LDCVs. The combination of patch-clamping with TIRF-Microscopy allows us to utilize one of the inherent advantages of whole-cell patch-clamping, i.e. tight control of the intracellular milieu, while simultaneously imaging the mobility of single secretory vesicles in real-time.

## Materials and Methods

### Cells, labeling and solutions

Bovine chromaffin cells were isolated, cultured as described [27] and used within 2–8 days after preparation.

For most patch-clamp measurements the bath solution was (in mM): 146 NaCl, 2.4 KCl, 10 HEPES, 1.2 MgCl_2_, 2.5 CaCl_2_, 10 glucose and 10 NaHCO_3_ (pH 7.4, 310 mOsm). The internal solution contained (in mM): 160 Cs-aspartic acid, 10 HEPES, 1 MgCl_2_, 2 Mg-ATP, 0.3 Na_2_-GTP (pH 7.2, 300 mOsm). Cells were depolarized to −5 mV from a holding potential of −70 mV, 7 times at 5 Hz for 100 ms.

For patch-clamp measurements in which secretion was triggered by flash photolysis of caged Ca^2+^ the bath solution was (in mM): 144.5 NaCl, 2.4 KCl, 10 HEPES, 4.0 MgCl_2_, 1.0 CaCl_2_ and 10 glucose (pH 7.5, 310 mOsm). The internal solution contained (in mM): 95 Cs-glutamate, 30.5 HEPES, 2 Mg-ATP, 0.2 Na_2_-GTP, 5 nitrophenyl-EGTA (NP-EGTA), 4.25 CaCl_2_, 0.4 Fura 4F and 0.4 Furaptra (pH 7.2, 300 mOsm).

Vesicles were stained by overexpressing NPY fused to monomeric DsRed (mRFP) or to Venus. The NPY construct, which was verified by DNA sequencing, was overexpressed by electroporation (Gene pulser II, Biorad, Hercules, CA, USA) of either NPY in pDsRed-N1-monomer (Clontech, Palo Alto CA, USA) or of NPY in Venus (kindly provide by Shigeo Takamori).

### Membrane capacitance recording

Conventional whole-cell recordings were performed with 3–5 MΩ pipettes and an EPC-9 patch-clamp amplifier controlled by PULSE software (Heka Elektronik, Lambrecht, Germany). For the generation of step-wise increases in [Ca^2+^]_i_, short flashes of ultraviolet light from a Xenon arc flash lamp (Rapp OptoElectronics, Hamburg, Germany) were applied to the cell. [Ca^2+^]_i_ was measured with a mixture of two ratiometric indicator dyes, Fura-4F [K_d_ (Ca^2+^): 0.77 mM] and Furaptra [K_d_ (Ca^2+^): 25 mM]. The dyes were excited with light alternating between 350 and 380 nm with a monochromator-based system (T.I.L.L. Photonics, Gräfelfing, Germany), and the fluorescent signal was measured with the camera (see below). An in vivo calibration curve was used to convert the ratio R of the fluorescent signals at both wavelengths into [Ca^2+^]_i_. The resting [Ca^2+^]_i_ before the flash was between 200–400 nM.

### Evanescent-wave imaging

The TIRF setup for most experiments was based on an IX70 microscope (Olympus) equipped with a 100×/1.45 NA Plan Apochromat Olympus objective, a TILL-TIRF condenser (TILL-Photonics, Gräfelfing, Germany) and a solid-state laser 85 YCA emitting at 561 nm (Melles Griot, Carlsbad, CA, USA). Images were acquired with a Micromax 512BFT camera (Princeton Instruments Inc., Trenton, NJ, USA) controlled with MetaMorph (Visitron, Puchheim, Germany). The acquisition rate was 5, 10 or 20 Hz and the exposure time was 50−100 ms. Pixel size was 130 nm and the penetration depth was 263 nm. For experiments in which secretion was induced through flash phololysis of caged Ca^2+^ the setup was based on an Axiovert 200 (Zeiss, Göttingen, Germany) equipped with a Fluar 100×/1.45NA Zeiss objective, a Zeiss TIRF slider and the solid-state laser 85 YCA emitting at 561 nm. The camera was an Andor iXon^EM^ (Belfast, Northern Ireland) controlled by software written in house based on Labview (National Instruments, München, Germany). The acquisition rate was 16 Hz and the exposure time was 50 ms. Pixel size was 150 nm and the penetration depth was 267 nm.

### Image analysis

Secretion of a vesicle was detected by a rapid reduction of the fluorescent signal from its own maximum value to the background level in less than 200 ms. Often this decrease of the fluorescent intensity of the vesicle was accompanied by a lateral spread of the fluorescent marker in the extracellular space, seen as a cloud of NPY-mRFP. In the experiment in which NPY-Venus was used to label the vesicles secretion was detected by a fast decay of fluorescent of the vesicle preceded by a brief transient increase of the fluorescent intensity. This increase in fluorescence was due to unquenching of the pH sensitive Venus.

### Chemicals

All the salts were from Sigma or Merck, PMA was from Sigma and the Ca^2+^ indicators (Fura 4F and Furaptra) were from Invitrogen (Karlsruhe, Germany). NP-EGTA was supplied by G. Ellis-Davies (MCP Hahnemann University, Philadelphia, PA).

### Data analysis

Data are represented as mean±S.E.M. Significance was assessed using a Mann-Whitney rank sum test since the data was not normally distributed. * represents a P value <0.05, **<0.01 and ***<0.005. Correlation was assessed using a Spearman rank order correlation test as the data set failed the Kolmogorov-Smirnov normality test.

## Supporting Information

Movie S1 TIRF-Microscopy (top) and membrane capacitance (bottom) recording of the cell shown in Figure 1a. This cell was stimulated for 2 s by depolarizing pulses and is shown 4 s prior to and 8.5 s after stimulation. Secretion of vesicles seen as a sharp decrease of fluorescence of individual spots (highlighted by white arrows) occurs simultaneously to the increase in membrane capacitance and only during the stimulation period (marked in red). Before and after stimulation vesicles may enter or leave the evanescent field as can be seen by a slow increase or decrease of individual fluorescent spots. Acquisition and display rate are 5 Hz.
(8.79 MB AVI)
